# Psycho-oncology: History, Current Status, and Future Directions in Japan

**DOI:** 10.31662/jmaj.2018-0001

**Published:** 2018-09-28

**Authors:** Tatsuo Akechi

**Affiliations:** 1 Department of Psychiatry and Cognitive-Behavioral Medicine, Nagoya City University Graduate School of Medical Sciences, Nagoya, Japan; 2 Department of Psycho-oncology and Palliative Medicine, Nagoya City University Hospital, Nagoya, Japan

**Keywords:** psycho-oncology, depression, anxiety, delirium, mind and body, Cancer Control Act

## Abstract

One of the most relevant risk factors for cancer is aging; thus, the number of patients who develop cancer and die is increasing in Japan. Cancer has been a leading cause of death since 1981, and more than one-fourth of Japanese people die of cancer. More than 1,000,000 and 37,000 Japanese people develop cancer and die every year, respectively, making it a major health problem in Japan.

Psycho-oncology is a relatively new medical field that was established in the 1970s in Western countries and introduced in Japan in the 1980s. Psycho-oncology was developed for investigating two issues neglected in previous medical research: the impact of behavioral and psychosocial factors on cancer morbidity and mortality and the psychological influence of cancer on patients, their families, and medical staff.

Because of progress made in cancer treatment, cancer diagnosis is not necessarily equivalent to a death sentence. However, approximately half of patients with cancer die, and many patients with cancer and their families need appropriate care for psychological distress. The most common psychiatric problems patients with cancer experience are adjustment disorders, major depression, and/or delirium. In addition, the suicide rate in Japan for individuals within 1 year of a cancer diagnosis is more than 20 times higher than that for individuals without cancer. Physical symptoms, such as pain and nausea/vomiting, can be closely associated with psychological function. Mental health professionals, particularly psycho-oncologists, are expected to work with other cancer professionals to manage patients' distress.

The present review focuses on patients with cancer' psychological distress and physical symptoms that are closely associated with psychological function and provides an overview of the current status of psycho-oncology in Japan. The future perspective of psycho-oncology is also discussed.

## Introduction

As one of the most relevant risk factors for the development of cancer is aging, and more than half of all new cancers occur in elderly people, the number of patients in Japan who develop cancer and who die from cancer continues to increase. Japan presently has the longest life expectancy at birth worldwide, and cancer has been the leading cause of death since 1981; more than one-fourth of Japanese people die from cancer ^(1)^. More than 1,000,000 and 37,000 Japanese people develop cancer and die every year, respectively. Thus, cancer is considered a major public health concern in Japan.

Traditionally, cancer diagnosis was equivalent to death sentence owing to a lack of effective treatment. However, recent progress made in cancer treatment has led to improvements in cancer survival. However, approximately half of the patients still die from cancer. The potentially life-threatening nature of the disease can cause profound distress among patients with cancer, and many experience psychological distress that requires appropriate care.

Increasing attention has been paid to psycho-oncology and palliative care in clinical oncology practice, particularly since truth-telling practices became more prevalent in Japan. Psycho-oncology was developed in Western countries in the 1970s for investigating two issues that had been neglected in previous research: the impact of behavioral and psychosocial factors on cancer morbidity and mortality and the psychological influence of cancer. Psycho-oncology is a relatively new medical field that was introduced in Japan in the 1980s. Psycho-oncology not only addresses the emotional aspects of cancer in patients and their families but also scientifically investigates the role of mind-body connection in cancer.

The purpose of this paper was to review the history and current status of psycho-oncology in Japan and to discuss the future direction of this medical field.

## History of Psycho-oncology

Psycho-oncology was introduced to clinical oncology practice in the 1970s, when truth-telling practices became prevalent in cancer care in Europe and the United States. In 1986, the Japanese Society of Clinical Psycho-Oncology (since renamed the Japan Psycho-Oncology Society) was established, and the first academic conference was held in 1987. This was the birth of psycho-oncology in Japan. Since then, psychiatry and psycho-oncology departments have gradually been established in specialized cancer hospitals ^(1)^.

In 2002, healthcare services provided by palliative care teams that included psychiatrists were covered by public health insurance in Japan. In 2007, the National Cancer Control Act was implemented, which states that palliative care (including comprehensive management of both psychosocial and physical distress) should be provided from the time of diagnosis of the disease. One of the most important aims of the Act is to improve the quality of life of patients with cancer and their families in Japan. The importance of the participation of psychiatrists, psychosomatic physicians, and clinical psychologists in cancer care has been clearly described in the Basic Plan to Promote Cancer Management. This attempt to make psychiatrists essential members of palliative care teams is a unique strategy worldwide and encourages the incorporation of psycho-oncology experts into medical oncology teams. In response to these efforts, the number of institutions that have set up palliative care teams in Japan has rapidly increased, and the involvement of mental health specialists (including psychiatrists, psychosomatic physicians, and psychologists) in cancer treatment has been extensively promoted.

Psycho-oncology experts have, therefore, become essential members of clinical oncology teams, particularly palliative care teams, and the 3^rd^ Basic Plan to Promote Cancer Management, implemented in 2017, introduced and clearly defined the role of a psycho-oncologist as “a psychiatrist or psychosomatic physician who is familiar with the influence of cancer on the minds of patients, families, and medical staff and engaged in ameliorating psychosocial distress in a clinical practice.”

As mentioned above, the application of psycho-oncology is legally supported in Japan. Most importantly, there is a recognition that patients and their families have profound psychosocial requirements. In the present article, I wish to outline why psycho-oncology is important in oncology practice, primarily focusing on the needs of patients and families and the role of psycho-oncologists. I also discuss future perspectives of psycho-oncology in Japan.

## Psychological Distress Experienced by Patients with Cancer

**Figure 1.**
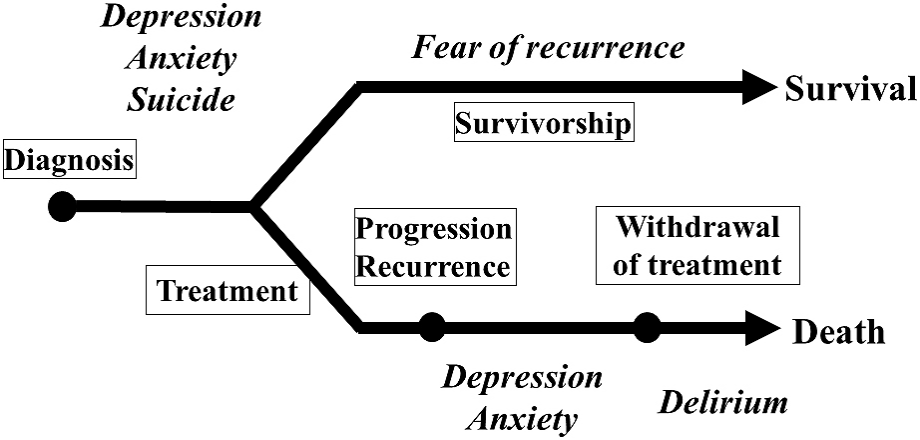
Cancer trajectory and psychological distress.

Patients with cancer can experience numerous kinds of psychological distress and/or psychiatric disorders during their illness trajectory, most commonly depression, anxiety, and delirium (Figure 1) ^(2), (3)^. The quality of life of patients with cancer is influenced not only by their malignant disease but also by comorbid medical and psychological conditions ^(4)^.

### 1) Depression/anxiety

Patients with cancer with clinical depression and anxiety are often diagnosed with adjustment disorders and major depression. In a meta-analysis of 70 oncological and hematological studies (10,071 individuals across 14 countries), Mitchell et al. reported that the prevalence of major depression was 15% (95% confidence intervals: 12-18), and the prevalence of adjustment disorders was 19%. The findings of the meta-analysis also showed a prevalence of 14% for major depression and 15% for adjustment disorders in 24 palliative care studies (4007 individuals across 7 countries) ^(5)^. Thus, the best estimate is that major depression has a point prevalence of 10%-20% in patients with cancer irrespective of cancer stage. This prevalence is similar to that found in patients with other chronic illnesses. The fear of recurrence experienced by cancer survivors has also received attention recently, as this is a very common and distressing symptom.

Appropriate management of this distress is essential for maintaining optimal cancer care, as anxiety and depression can lead to serious and far-reaching negative consequences in patients with cancer. These psychological problems can reduce patients' quantity and quality of life and cause severe suffering and suicide, as well as poor adherence to anticancer treatment and psychological distress in family members ^(6), (7), (8), (9)^. Even though both psychotherapies and pharmacotherapies are useful treatments for anxiety and depression among patients with cancer ^(10), (11), (12)^, there is evidence that distress, particularly depression, is often overlooked by oncology staff ^(13)^. Therefore, the management of depression and anxiety remains a problem in oncological care in Japan.

### 2) Delirium

Delirium is a frequently occurring and serious clinical problem, particularly for hospitalized advanced patients with cancer, and is associated with an increased risk of morbidity and mortality and increased healthcare costs, such as those associated with prolonged hospitalization ^(14), (15)^. The prevalence of delirium in hospitalized patients, including patients with cancer, ranges from 10% to 30% ^(16), (17)^. Among advanced patients with cancer who are hospitalized, the prevalence of delirium ranges from 30% to 40% even at the time of admission, and up to 90% of advanced patients with cancer receiving end-of-life care develop delirium ^(14), (16)^. There are several delirium subtypes, and the disorder is now classified into three main subtypes as follows: hyperactive, hypoactive, and mixed. Hypoactive delirium, which is characterized by sluggishness and lethargy, is the most common type, and is also associated with profound distress in patients with cancer and their families although extreme agitation is not observed in hypoactive delirium ^(18)^.

The treatment of delirium generally includes the identifying and addressing of underlying causes and the concurrent management of delirium symptoms using both non-pharmacological and pharmacological strategies ^(19)^. However, no standard management strategies exist, and there is a particular lack of evidence of effective pharmacotherapies, for hypoactive delirium.

### 3) Suicide

Previous epidemiological studies and meta-analyses show that the suicide rate of patients with cancer is approximately twice as high as that of the general population ^(20), (21)^. Findings of recent studies support this pattern. For example, one Western study that investigated the effect of cancer diagnosis found that diagnosis can produce acute stress associated with higher suicide rates, particularly in the first weeks, and that these effects last for at least 6 months after cancer diagnosis ^(22), (23)^. In addition, a Japanese study we conducted revealed that the suicide rate for people within 1 year of a cancer diagnosis is more than 20 times higher than that for people not diagnosed with cancer ^(22)^. These studies suggest that intervention immediately after cancer diagnosis is critical for preventing suicide among patients with cancer. However, the underlying reasons as to why patients with cancer are more likely to commit suicide very soon after cancer diagnosis are not clearly understood. Furthermore, previous studies consistently indicate that males, patients suffering from advanced stage cancer at the time of diagnosis, and patients with head and neck cancer are more likely to commit suicide ^(24), (25)^. As mentioned above, another common risk factor of suicide is being in the first 2 or 3 months after a cancer diagnosis ^(21), (25), (26)^.

Remarkably, psychological autopsy studies of patients with cancer who committed suicide suggest that the most frequent underlying cause is depression, not physical pain ^(27)^. Filiberti et al. examined five patients with terminal cancer who had committed suicide while receiving palliative home care in Italy. They found that majority of the patients had experienced both physical suffering and psychological distress, including depression. They indicate that all the patients experienced the loss of autonomy and independence and refused to be a burden to others ^(28)^. Some researchers claim that there may be a rational basis to suicide among some patients with terminal cancer ^(29)^.

Compared with other developed countries, Japan has a remarkably high suicide rate. Some researchers have proposed that core cancer hospitals should conduct research on suicide prevention and that psycho-oncology experts could play an important role in suicide prevention efforts in Japan ^(30)^.

## Physical Symptoms Closely Associated with Psychological Function

During the illness trajectory, most patients with cancer experience several kinds of physical distress. Some of these symptoms are closely associated with psychological function.

### 1) Pain

More than half of patients with cancer experience pain during their illness trajectory. The International Association for the Study of Pain defines pain as “an unpleasant sensory and emotional experience associated with actual or potential tissue damage, or described in terms of such damage ^(31)^.” Pain is a multidimensional symptom that involves both physical and psychosocial distress. Thus, terms such as “psychogenic pain,” which implies that pain is purely psychological, do not adequately reflect the complex nature of the pain. Appropriate pain management should include both analgesia and individualized psychosocial care that focuses on patients' psychological experiences of pain. There are numerous opportunities to offer patients with cancer multidisciplinary treatment for pain management, and multidisciplinary teams usually include mental health professionals who have a good understanding of the multifaceted aspects of pain. Pain and psychological distress, including anxiety and depression, have a bidirectional association ^(32)^.

What kind of approach is required to appropriately assess and manage patients with cancer who suffer from pain? When pain is clearly caused by the progression of advanced cancer, drugs such as non-steroidal anti-inflammatory analgesics, weak opioids, and strong opioids are used, as appropriate, in pharmacological therapy. In the case of neuropathic pain, which is often refractory to the usual pharmacotherapies, psychotropic agents such as antidepressants and anticonvulsants are often used in combination with the above-mentioned drugs ^(33)^. These administrative methods are described in the World Health Organization's cancer pain treatment ladder ^(34)^. Cancer pain has mostly physical causes, but chronic pain in cancer survivors who do not have active cancer has gained recent attention. There are no clear findings regarding the prevalence of pain in cancer survivors; however, at least 5%-10% of cancer survivors suffer from distressing chronic pain, which is particularly common in breast patients with cancer ^(35)^. These patients are also likely to have psychiatric problems, such as anxiety and depression. Therefore, psycho-oncologists play an important role in evaluating concurrent psychosocial issues and advising patients how to manage them. Numerous patients feel that the development of pain is a sign of recurrent cancer or disease progression. In such cases, in addition to alleviating the physical pain, psychological support is crucial.

Advanced/terminal patients with cancer often develop cognitive impairment, such as delirium. In such cases, the expression of pain may be amplified because of the lack of emotional suppression owing to delirium. When the expression of pain in advanced and/or terminally ill patients with cancer is unstable or ambiguous, and when it results from delirium, appropriate management of delirium by psycho-oncologists can sometimes help in decreasing pain intensity ^(32)^.

### 2) Anticipatory nausea and vomiting

Nausea and vomiting that often accompany later cancer treatments start even prior to the administration of the chemotherapeutic agent; this phenomenon has been defined as anticipatory nausea and vomiting (ANV) ^(36)^. ANV can best be understood in terms of classical conditioning. Typically, ANV is a learned response to one or more distinctive features of the chemotherapy clinic (conditioned stimuli) associated with the administration of emetogenic chemotherapy (unconditioned stimuli) ^(37)^.

Recent advances in supportive therapy for preventing chemotherapy-induced nausea and vomiting (CINV), including the addition of corticosteroids to 5-HT3 receptor antagonists and/or neurokinin 1-receptor antagonists, has improved the management of CINV ^(38), (39)^, and this has substantially altered patient perceptions of the side effects of chemotherapy. For example, earlier studies of patient perceptions of the side effects of cancer chemotherapy in the 1990s repeatedly demonstrated that CINV was one of the most significant and distressing symptoms for patients with cancer receiving chemotherapy ^(40)^. On the contrary, a similar report in 2002 showed a marked change and indicated that predominant concerns were fatigue and psychosocial quality of life rather than CINV ^(41)^. However, several studies have shown that CINV, including anticipatory nausea, remain a significant problem for patients receiving moderately or highly emetogenic regimens, even after treatment using 5-HT3 receptor antagonists and corticosteroids, and that physicians and nurses may underestimate the risk of delayed CINV ^(42)^. Previous studies indicate that approximately 30%-60% of patients experience anticipatory nausea, although the occurrence rate differs according to several factors, such as the type of chemotherapy, post-chemotherapy vomiting, age, gender, and anxiety level ^(43), (44), (45)^. Because once ANV develops, it is sometimes challenging to control and often persists for up to a year ^(46)^, this is still a critical problem for patients with cancer who need highly emetogenic chemotherapeutic regimens ^(47)^.

Behavioral treatments for ANV include systematic desensitization and pharmacotherapies such as benzodiazepines; typical antiemetic agents are not effective for ANV ^(48)^. Thus, the appropriate assessment and management of ANV are essential, particularly for patients with cancer who are treated using chemotherapeutic agents.

## Care for Family Members and/or Bereaved Families

The experience with cancer is generally assumed to cause considerable stress, not only to patients but also to caregivers who share the patient's distressing illness trajectory. Family members most frequently fulfill the role of primary care providers for patients with cancer. In Japan, treatments for medical diseases such as cancer are increasingly provided on an outpatient basis; thus, much of the burden of care has shifted from healthcare professionals to patients and their families.

Previous Western studies suggest that approximately 10%-30% of family members experience some form of psychiatric morbidity ^(49)^, but there have been very few studies regarding this in Japan. It is established that the death of a close family member is one of the most stressful life events ^(50)^. However, there are cross-cultural differences in the function of the family between Western and Asian countries ^(51)^. For example, family opinions are accorded a larger role by Japanese patients than by American patients, and this may affect decision-making processes, such as disclosure of an incurable cancer diagnosis ^(52)^. These data suggest that family members in Japan may experience a different type of psychological distress and/or burden than those in other countries, owing to cultural differences and the way the Japanese medical system manages patients with cancer. However, some previous Japanese studies show that adjustment disorders and major depression are most common in families of patients with cancer ^(53), (54)^, and these findings are consistent with Western study findings. It is interesting that these disorders are the most common psychiatric disorders among patients with cancer. These findings suggest that both patients with cancer and their families, as a socially integrated human unit, experience similar psychological distress during the illness trajectory ^(55)^.

Thus, good cancer care must include care for family members, and the Cancer Control Act emphasizes the care needs of the whole family. However, one Japanese study has shown that very few family members are provided with psychiatric support and/or treatment in Japan ^(54)^. There is no doubt that support for family members will become more essential; therefore, the development of a comprehensive support system for caregivers of patients with cancer is an urgent issue in clinical oncology in Japan. Cancer should be treated as a family issue and a family problem. Family members should be considered as “second order patients ^(56)^.”

## A Good Death for Japanese Patients with Cancer

One of the most important goals of palliative care, particularly end-of-life care, is achieving a “good death” for the patient. Several previous studies have discussed the concept of a good death. For example, Steinhauser et al. reported that factors such as pain and symptom management, preparation for death, achieving a sense of completion, decisions about treatment preferences, and being treated as a “whole person” are important for patients in the United States ^(57)^. Similarly, a qualitative Japanese study identified 18 aspects of a good death, including “physical and psychological comfort,” “dying in a favorite place,” and a “good relationship with medical staff” (Table 1) ^(58)^. The Japanese findings demonstrate that medical staff should recognize broader good death concepts beyond symptom control. In addition to symptom management (e.g., physical and psychological comfort) and improvements in the healthcare system (e.g., dying in a favorite place), challenging psycho-existential issues (e.g., hope and pleasure, not being a burden to others and “completion of life”) are an essential part of quality palliative care.

**Table 1.** Components of Good Death in Japan.

Constantly important domain	1.Physical and psychological comfort
	2. Dying in a favorite place
	3. Good relationship with medical staff
	4. Maintaining hope and pleasure
	5. Not being a burden to others
	6. Good relationship with family
	7. Physical and cognitive control
	8. Environmental comfort
	9. Being respected as an individual
	10. Life completion
Individually different domain	11. Natural death
	12. Preparation for death
	13. Role accomplishment and contribution to others
	14. Unawareness of death
	15. Fighting against cancer
	16. Pride and beauty
	17. Control over future
	18. Religious and spiritual comfort

Constantly important domain: Most Japanese endorse the relevance as component of good death; Individually different domain: Some Japanese endorse the relevance as component of good death but others do not.

## Psychosocial Factors and Cancer Incidence and Survival

There has been substantial and continuous interest in whether factors such as human behavior style, personality traits, psychosocial factors, and life events affect the incidence of cancer and survival time among patients with cancer. Some previous studies have shown that people with certain personality traits are more susceptible to develop cancer and that coping styles following cancer diagnosis can influence longevity and survival ^(59), (60)^. However, some of this research was characterized by weak study designs, and more rigorous studies have since been conducted to replicate the findings ^(61)^. However, many of the previous findings have not been adequately replicated. Therefore, even though there is a substantial body of research on the associations between psychosocial factors and cancer outcomes, the findings are inconsistent ^(62), (63)^. A 2008 meta-analysis suggested that stress-related psychosocial factors have an adverse effect on cancer incidence and survival, although the analysis found evidence of publication bias, and the results should be interpreted with caution ^(64)^. Because there is still great interest among the general population in the association between cancer outcomes and psychosocial factors, psycho-oncology is expected to clarify the relationship between these factors.

## Future Perspectives

As mentioned above, more than 1 million Japanese people are diagnosed with cancer every year, and the number of cancer survivors is on the rise. Considering the large number of cancer survivors and their family members, and the fact that many of them experience psychological distress, psycho-oncology plays an essential role in improving the quality of life of patients and their families. However, several issues need addressing, such as the insufficient number of psycho-oncologists, lack of high quality evidence about prevention, early detection, and treatment of psychological distress experienced by patients with cancer and their families while a recent Japanese study suggests the efficacy of oncologists' communication skills training on prevention for psychological distress among patients ^(65)^. It is not easy to overcome cancer, but further developments in psycho-oncology may help individuals to live a fulfilling life even after they have been diagnosed with cancer.

## Article Information

### Conflicts of Interest

Tatsuo Akechi received lectures fees from AstraZeneca, Daiichi Sankyo Co. Ltd., Sumitomo Dainippon Pharma Co., Ltd., Eizai, Hisamitsu Pharmaceutical Co. Inc., Eli Lilly and Company, MSD Pharmaceuticals, Meiji Seika Pharma Co. Ltd, Mochida Pharmaceutical Co. Ltd., Pfizer Inc., Novartis, Otsuka Pharmaceutical Co. Ltd., Shionogi & Co. Ltd., Takeda Pharmaceutical Co. Ltd., Mitsubishi Tanabe Pharma Corporation, Terumo Corporation, and Yoshitomi Pharmaceutical; royalties from Igaku-Shoin Ltd., Kagakuhyoron-sha Publishing Co., and Seiwa Shoten; and research funds from Daiichi Sankyo Co. Ltd., Eizai, MSD Pharmaceuticals, Pfizer Inc., Novartis, and Mitsubishi Tanabe Pharma Corporation.

### Acknowledgement

We thank Edanz Group (www.edanzediting.com) for editing a draft of this manuscript.
